# The Swallowing Clinical Assessment Score in Parkinson's Disease (SCAS-PD) Is a Valid and Low-Cost Tool for Evaluation of Dysphagia: A Gold-Standard Comparison Study

**DOI:** 10.1155/2019/7984635

**Published:** 2019-03-13

**Authors:** Larissa L. Branco, Sheila Trentin, Carla Helena Augustin Schwanke, Irenio Gomes, Fernanda Loureiro

**Affiliations:** ^1^Graduate Program in Biomedical Gerontology, School of Medicine, Pontifical Catholic University of Rio Grande do Sul, Porto Alegre, Brazil; ^2^Department of Neurology, São Lucas Hospital, Pontifical Catholic University of Rio Grande do Sul, Porto Alegre, Brazil

## Abstract

**Background:**

Dysphagia is a predictor of mortality in Parkinson's disease (PD). Developing alternative methods to videofluoroscopy swallowing study (VFSS) for the evaluation of dysphagia is a public health necessity. The *Swallowing Clinical Assessment Score in Parkinson's Disease (SCAS-PD)* is an alternative and low-cost tool for diagnosis of dysphagia, but had not been properly validated in comparison to the gold-standard method. The objective of this study was to assess the validity and reliability of the SCAS-PD.

**Methods:**

SCAS-PD was applied to 31 patients with PD, and VFSS was conducted concurrently. This clinical assessment uses different volumes and viscosities to identify signs of swallowing impairments. For validation purposes, the interclass correlation coefficient and the weighted kappa were calculated. The AUC of the ROC curve, sensitivity, and specificity values for detection of penetration/aspiration (PA) were assessed. Internal consistency was calculated by Cronbach's alpha.

**Results:**

Fifty-one percent of patients were classified with dysphagia. SCAS-PD was differentiated between normal/functional deglutition and dysphagia with AUC 0.97, 95% CI 0.92–1.00, and an optimal cutoff at 19 (sensitivity 100% and specificity 87.5%). The internal consistency was *α* = 0.91 for the total score. The internal consistency of the SCAS-PD domains was oral phase (*α* = 0.73), pharyngeal phase (*α* = 0.86), and signs of PA (*α* = 0.95). The weighted kappa analysis demonstrated a high rate of concordance at 0.71 (*p* < 0.001) between SCAS-PD and VFSS.

**Conclusions:**

SCAS-PD has been shown to have a good concordance with the VFSS. Considering this, SCAS-PD is highly applicable in clinical settings, since it is a simple and low-cost diagnostic tool for detecting dysphagia in PD patients.

## 1. Introduction

Dysphagia is a predictor of mortality in Parkinson's disease (PD). Respiratory failure as a result of dysphagia and aspiration is considered as the main cause of death among PD patients [1]. Furthermore, dysphagia can be nonsymptomatic, characterized as silent aspiration [2].

Despite the existence of good screening questionnaires [3, 4] to detect dysphagia in this population, the clinical evaluation of swallowing remains a challenge in the absence of objective tests. The videofluoroscopy swallowing study (VFSS) [5] is considered the gold standard of diagnostic methods for the detection of dysphagia. However, this test requires an adequate posture from the patient, collaboration, ingestion of barium, and exposure to X-ray. Due to these factors, it is not indicated for every patient complaining of dysphagia. Furthermore, the VFSS requires a high-cost equipment that is not available at every health clinic and a health professional trained to conduct the examination.

One can verify in the literature that although cervical auscultation [6], changes in vocal quality [7], and respiratory pattern [8] are broadly used as clinical signs for aspiration, there are few studies presenting data on the accuracy and contribution of these techniques for the detection of dysphagia in clinical practice.

A clinical assessment for dysphagia must be proposed, especially considering the peculiarities of the clinical conditions. Depending on the etiology, the signs suggesting aspiration may not be the same. Parkinsonian symptoms are responsible for inefficiency of the oral-pharyngeal mechanisms of swallowing, decreasing the excursion of the hyolaryngeal complex and lacking of coordination in swallowing and breathing [9–12]. However, parameters such as an alteration in vocal quality may not be good for detecting dysphagia in these conditions [13].

A protocol for the clinical evaluation of swallowing that uses different volumes and consistencies of food can serve as an excellent tool for detecting dysphagia, with a low cost and simple application, and can be applied in settings outside of hospitals.

The *Swallowing Clinical Assessment Score in Parkinson's Disease (SCAS-PD)* [14] was developed to assess dysphagia in PD patients and consists of 12 items that identify the occurrence of alterations in the oral and pharyngeal phases of swallowing.

The objective of this study was to validate SCAS-PD in comparison to the videofluoroscopy swallowing study (VFSS), verify its internal consistency, and establish cutoff points to stratify the severity of dysphagia.

## 2. Methods

### 2.1. Population and Sample

Thirty-one individuals with idiopathic PD were recruited. To calculate the sample size, an 80% frequency of signs of dysphagia in PD patients was taken into account, considering an intraclass correlation coefficient above 0.7 with a level of significance of 5% and power of 80%, leading to a minimum sample of 31 individuals. The items in STARD 2015 for diagnostic accuracy were followed [15].

The individuals included had a PD diagnosis, were treated at the Neurology Clinic of the São Lucas Hospital at Pontifical Catholic University of Rio Grande do Sul, Brazil, presented swallowing complaints according to their answer to question seven in the unified Parkinson's disease rating scale (UPDRS) [16]. Excluded patients had other associated neurological diseases, such as vascular accidents, cranioencephalic trauma, severe cognitive impairments, or other impairments that cause difficulties in mobility and in the sensitivity of the oral-pharyngeal region. SCAS-PD was applied concurrently with the VFSS.

All patients had the clinical state of their disease evaluated by neurologists specializing in movement disorders, in accordance with the Hoehn and Yahr Stages of Parkinson's Disease Scale [17] and the UPDRS part III (UPDRS) [16]. Cognition was evaluated through the Montreal Cognitive Assessment (MoCA) [18] using a cutoff point of 18 in order to exclude participants with severe cognitive impairment. Since the participants had a low level of education, the cutoff point between 20 and 25 on MoCA does not indicate significant cognitive impairment in this population.

### 2.2. Ethical Aspects

The project was approved by Institution's Ethics Committee (report number 1.837.116). All participants signed the Terms of Free and Explained Consent, which was conducted according to the Regulatory Norms and Regulations for Research involving Humans (Resolution 466/12), and were informed of the study's objectives, processes to be conducted, and guarantees.

### 2.3. SCAS-PD Protocol

SCAS-PD is a clinical assessment consisting of 12 items that identify the occurrence of specific alterations in the oral and pharyngeal phases of swallowing [14] (see Table 1). The protocol is conducted using three consistencies of food: 20 ml liquid (water), 10 ml paste (liquid with pudding consistency using the Thick & Easy food thickener), and one unit of solid (salt cracker). Application of SCAS-PD lasts approximately five minutes, since it involves solely observing the three swallows.

This assessment has a range of 0 to 354 points, where the sum of the points can suggest symptoms of dysphagia. A score was attributed to each item according to its relevance in the literature. The following aspects are evaluated in the oral phase: prehension of food (1.0 point), labial discharge (1.0 point), oral transit time (2.0 points), and presence of residue (2.0 points). The oral phase can reach a maximum score of 18 after the three evaluated swallows. The following items are evaluated during the pharyngeal phase: multiple deglutition (2.0 points), laryngeal elevation (10 points), and cervical auscultation (10 points), with a maximum total of 66 points. The signs of laryngotracheal PA that make up the SCAS-PD test are throat clearing (10 points), cough (15 points), change in vocal quality (15 points), choking (20 points), and respiratory impairment (30 points), with a maximum total of 270 points.

During the oral phase, prolonged oral transit was considered to be longer than four seconds. Presence of residue refers to any food residue left anywhere in the oral cavity after swallowing. The triggering of the oral and pharyngeal phases of swallowing is verified through cervical auscultation, a process through which one can hear the sounds of swallowing by using an amplification instrument, such as a stethoscope. This aids the evaluation of the pharyngeal phase of swallowing through an attempt to determine the integrity of the air passage's protection mechanism and by establishing the length of the sounds associated with swallowing. For this study, a Littmann Classic II Pediatric stethoscope was used during the swallowing of all volumes and consistencies, and any alterations before, during, or after swallowing were noted. A cervical auscultation was considered noisy if there were sounds during the respiration, swallow, and respiration sequence that had not been observed before the food was offered. Vocal quality was assessed after each swallow, since a wet voice can indicate a presence of food residue in the pharynx or vocal cords. The occurrence of throat clearing was taken into consideration when an attempt to clear food residue after swallowing was observed. The presence of involuntary coughing was also taken into consideration for each episode, independently of it happening before, during, or after swallowing. Choking is defined as a partial or complete obstruction of airflow, resulting from the entrance of a foreign body in the lower air passages, possibly leading to cyanosis or asphyxiation. Choking was considered when there was a quick recovery without the occurrence of cyanosis and when there was a quick recovery of the base respiratory frequency and the possible occurrence of cyanosis or with a difficult recovery of the base respiratory frequency. Respiration was evaluated after each swallow, with a change in the respiratory pattern being considering any event, such as a change in respiratory frequency, alteration in the coordination breathing/swallowing, dyspnea, and fatigue, for each food offering tested.

The SCAS-PD score was developed to stratify the levels of severity of dysphagia, with a preliminary cutoff defined as normal ≤2, functional swallowing as >2 ≤ 15, mild dysphagia as >15 ≤ 35, moderate dysphagia as >35 ≤ 60, and severe dysphagia as >60.

The researcher that conducted the clinical assessment was blind as to the results of the VFSS, which was conducted concurrently. The clinical assessment of all patients was conducted by the same evaluator, a speech pathologist specializing in dysphagia, while the VFSS was conducted and analyzed by a speech pathologist with 15 years of experience working with dysphagia.

### 2.4. Videofluoroscopy Swallowing Study

The VFSS was conducted in the Radiology Department of the São Lucas Hospital by the speech pathologist responsible for the Speech Pathology Rehabilitation department. The fluoroscopy equipment used was a *Siemens* model *Axion Iconos R100*, attached to a computerized image recording system, which allows for a detailed analysis of the test. During the VFSS, subjects remained seated, with the images being recorded from the lateral and anteroposterior positions and with the superior and inferior limits ranging from the oral cavity to the stomach. Participants in the study underwent the full examination, ingesting all consistencies offered. Only one patient with severe dysphagia was unable to ingest the solid consistency and received the maximum score for this consistency. The VFSS report was based on the Severity Scale Dysphagia [19]. Classification according to this scale is within normal pattern, within functional/spontaneous compensation patterns, discrete dysphagia, moderate dysphagia, and intense dysphagia.

### 2.5. Statistical Analysis

A database was created specifically for the purpose of conducting the statistical analysis in this study. Data were analyzed in the program SPSS version 21.0. For this study, intraclass correlation coefficient (ICC) and weighted kappa were used to establish the concordance between the variables for severity in the SCAS-PD and the Severity Scale Dysphagia for the VFSS. The area of the receiver operating characteristic (ROC) curve was calculated to verify the accuracy of SCAS-PD, and the sensitivity and specificity values in detecting PA of food in the larynx were verified. Finally, cutoff points that clinically stratify the levels of severity of dysphagia for SCAS-PD were revised with greater accuracy. In order to validate SCAS-PD, the internal consistency was also calculated using Cronbach's alpha, as well as for each domain (oral phase, pharyngeal phase, and signs of laryngotracheal PA).

Data were evaluated quantitatively, using descriptive analysis, median, and standard deviation. The level of significance adopted was 5% (*p* < 0.05).

## 3. Results

The sociodemographic characteristics of the participants and their dysphagia classification are presented in Table 2. According to SCAS-PD, 45% were classified as without alteration (normal/functional) and 55% with dysphagia. According to the VFSS, 51% were classified within normal/functional standards. SCAS-PD did not fail to identify any individual with dysphagia.

The ICC between the results of the SCAS-PD and the VFSS presented a good correlation, as seen in Table 3. The weighted kappa analysis demonstrated a high rate of concordance. The kappa analysis by severity of dysphagia found the following results: normal (good concordance), functional (moderate concordance), mild (good concordance), moderate (weak concordance), and severe (weak concordance).

**Figure 1:**
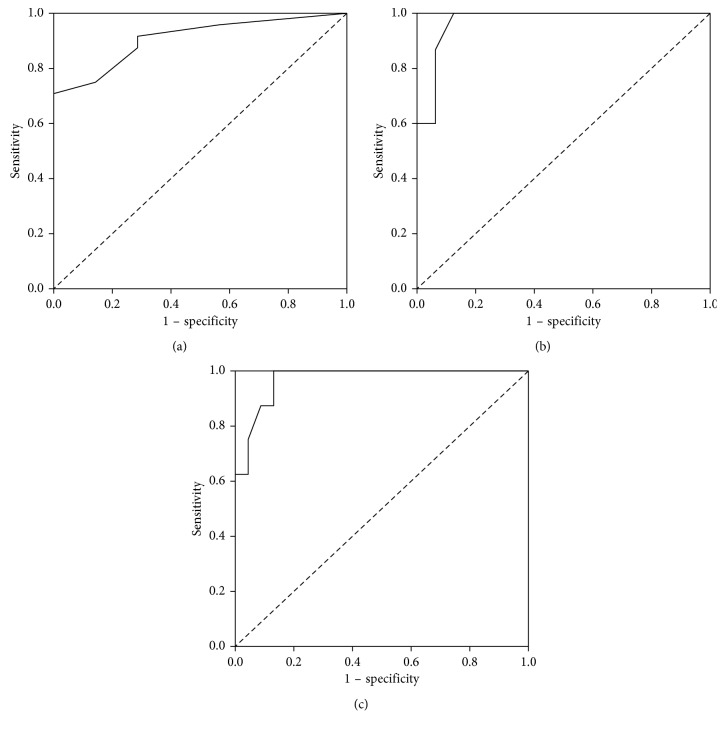
ROC curve for (a) swallowing evaluation at the functional level, (b) diagnosis of dysphagia at any level of severity (mild, moderate, and severe), and (c) moderate to severe dysphagia.

The accuracy of SCAS-PD and the ROC curves is presented in Figure 1, and the diagnostic proprieties are presented in Table 4. The different ROC curves were calculated in order to obtain the cutoff points stratified for each level of severity of dysphagia.

SCAS-PD's internal consistency was *α* = 0.91 for the total score. For the oral phase, *α* = 0.73; for the pharyngeal phase, *α* = 0.86; and for the signs of PA, *α* = 0.95. These results demonstrate a good reliability for SCAS-PD in all evaluated domains (*α* > 0.7).

## 4. Discussion

Early diagnosis for dysphagia can be difficult in PD, since patients may not report complaints and have little awareness of alterations in swallowing [2]. Due to this, there is a need for the development of a clinical assessment specifically for this population.

VFSS is the technique most frequently used to evaluate swallowing. Due to a variety of factors, such as cost and accessibility, the VFSS is not recommended for all patients. There is a need for a validated clinical assessment of dysphagia that identifies the signs of laryngotracheal aspiration in the absence of objective tests. SCAS-PD presented a good correlation in comparison to the VFSS based on the ICC analysis.

Kertscher et al. [20], following the Cochrane collaboration criteria for revising studies on dysphagia screening, identified only four studies focusing on patients with neurological impairments, out of a total of 14, that could be considered to have sufficient methodology. Of these, three were conducted on heterogeneous populations [21–23], and only one conducted the validation with a specific etiological group, in this case with poststroke patients [23]. Of the four studies highlighted in this review, only one applied three standardized consistencies using controlled volumes and thickening agents [24]. We believe that the variety of consistencies and volumes administered in SCAS-PD favors diagnostic accuracy and increased sensitivity in the clinical assessment of swallowing.

One study that attempted to validate the vocal quality parameter as a sign of aspiration in comparison to the VFSS found a low sensitivity and high specificity [25]. One of the limitations described by the authors was that the individuals included in the study presented different etiologies. Despite the limitation of our sample size, we believe that conducting the validation of our instrument on a specific neurological group (PD patients) increased its diagnostic accuracy.

Other studies conducted specifically on PD populations verified some clinical signs, such as coughing and alterations in vocal quality, in the detection of aspiration. Sampaio et al. [13] reported a limitation of vocal change as a clinical sign, pointing out that a lack of wet voice should be seen with caution and be associated with other clinical signs. Meanwhile, airflow measured in a voluntary cough had a high capacity to predict aspiration in this population [8]. SCAS-PD's proposal to consider signs suggestive of aspiration, such as alterations in breathing, presence of expectoration, coughing, and choking, in addition to the use of cervical auscultation and the verification of changes in vocal quality, resulted in a high sensitivity of 100% with a good specificity of 87.5%, demonstrating that a combination of clinical signs increases diagnostic accuracy. These results show that a negative evaluation by SCAS-PD indicates the absence of dysphagia.

We must also emphasize the fact that the clinical evaluation of swallowing was conducted concurrently with the VFSS in all individuals evaluated. Some studies consider it a limitation when the standard test and the index test are conducted at different times [25, 26].

The low concordance between SCAS-PD and VFSS for moderate severity, as seen in Table 3, is due to the low number of participants in the more advanced phases of the disease and of dysphagia. However, it is important to note that the clinical evaluation showed to be more conservative than the objective examination, classifying as severe cases that were considered moderate through the VFSS. To us, this aspect of the clinical evaluation is a positive, since the objective is to identify and prevent the risks of possible laryngotracheal aspiration. In our final proposal, a SCAS-PD score >35 points suggests a high risk of laryngotracheal aspiration.

SCAS-PD has been shown to be a clinical assessment of dysphagia in PD patients with good reliability. After validating SCAS-PD, the cutoff points for the levels of severity were defined as normal ≤3, functional >3 ≤ 19, mild >19 ≤ 35, and moderate to severe >35 points.

## 5. Conclusion

SCAS-PD has demonstrated that it is a good assessment tool for dysphagia in Parkinsonian patients, capable of detecting the clinical signs of laryngotracheal PA with a good concordance with a gold-standard VFSS. It is essential to the early detection of dysphagia and prevention of aggravations like malnutrition, dehydration, and bronchopneumonia.

One of the main limitations of this study was the small sample due to some difficulties, such as patients' accessibility to conduct the VFSS. However, since the participants were predominantly in the initial and mild phases of the disease, 51% presented dysphagia in the VFSS that was also detected through SCAS-PD; so, we believe it was possible to validate the instrument, whose main goal is to distinguish normal and functional swallowing levels from the states of dysphagia that present clinical signs of laryngotracheal PA.

SCAS-PD presented a good concordance with the VFSS. This clinical assessment has good applicability, consisting of offering three consistencies of food. The clinical assessment presented high sensitivity and a high positive predictive value for the diagnosis of dysphagia. It should be applied by trained speech pathologists capable of identifying the clinical signs of laryngotracheal PA that are a component of the tool. SCAS-PD is only one part of the evaluation of dysphagia, which should be global and multidisciplinary.

## Figures and Tables

**Table 1:** Score attributed to each sign of alteration in swallowing in the original SCAS-PD. Source: Loureiro et al. [14].

Signs suggesting alteration in swallowing found during assessment	Score	
	For each offering	Max. possible
Oral phase		
Altered lip closure	1.0	3.0
Labial discharge	1.0	3.0
Prolonged oral transit time	2.0	6.0
Residue	2.0	6.0
Total		**18.0**
Pharyngeal phase		
Multiple deglutition	2.0	6.0
Reduced larynx elevation	10.0	30.0
Altered cervical auscultation	10.0	30.0
Total		**66.0**
Signs of penetration/aspiration		
Throat clearing	10.0	30.0
Cough	15.0	45.0
Change voice quality	15.0	45.0
Choking	20.0	60.0
Alteration in breathing	30.0	90.0
Total		**270.0**
Total		**354.0**

**Table 2:** Demographic, cultural, and clinical characteristics of the sample.

Variables	*n*=31
Age (years), mean ± SD	68.8 ± 7.6
Gender, *n* (%)	
Male	14 (45.2)
Female	17 (54.8)
Education (years), median (min-max)	5 (3–7)
MoCA, mean ± SD	19.4 ± 5.4
Hohen and Yahr Scale, *n* (%)	
1	5 (16.1)
2	17 (54.8)
3	6 (19.4)
4	2 (6.5)
5	1 (3.2)
UPDRS part III, mean (min-max)	30 (1–65)
Duration of disease (years), median (min-max)	9 (6–12)
Severity of dysphagia	
SCAS-PD, *n* (%)	
Normal	7 (22.6)
Functional	5 (22.5)
Mild	6 (19.4)
Moderate	6 (19.4)
Severe	4 (16.1)
VFSS, *n* (%)	
Normal	6 (22.6)
Functional	8 (29.0)
Mild	7 (22.6)
Moderate	6 (22.6)
Severe	1 (3.2)

MoCA, Montreal Cognitive Assessment; UPDRS, unified Parkinson's disease rating scale; part III, motor examination; SCAS-PD, Swallowing Clinical Assessment Score in Parkinson's disease; VFSS, videofluoroscopy swallowing study.

**Table 3:** Correlation between SCAS-PD and VFSS according to the percentage of sample.

VFSS	SCAS-PD											
	Total sample		Severity of dysphagia									
			Normal		Functional		Mild		Moderate		Severe	
	CCI (*p*)	Weighted kappa (*p*)	*K*	*p*	*K*	*p*	*K*	*p*	*K*	*p*	*K*	*p*
Severity of dysphagia												
Total sample	0.935 (<0.001)	0.71 (<0.001)	—	—	—	—	—	—	—	—	—	—
Normal	—	—	0.63	<0.001	—	—	—	—	—	—	—	—
Functional	—	—	—	—	0.50	0.002	—	—	—	—	—	—
Mild	—	—	—	—	—	—	0.71	<0.001	—	—	—	—
Moderate	—	—	—	—	—	—	—	—	0.32	0.037	—	—
Severe	—	—	—	—	—	—	—	—	—	—	0.30	0.010

ICC, interclass correlation coefficient; k, kappa coefficient; SCAS-PD, Swallowing Clinical Assessment Score in Parkinson's disease; VFSS, videofluoroscopy swallowing study.

**Table 4:** Diagnostic properties of the Swallowing Clinical Assessment Score in Parkinson's disease (SCAS-PD).

Diagnostic properties/frequency	Severity of dysphagia		
	Functional	Any level of severity	Moderate to severe
Cutoff (points)	3	19	35
AUC (95% CI)	0.91 (0.80–1.00)	0.97 (0.92–1.00)	0.97 (0.92–1.00)
Sensitivity (%)	91.7	100	100
Specificity (%)	71.4	87.5	87.0

Any level of severity: mild, moderate, and severe; AUC: area under the curve; 95% CI: 95% confidence interval.

## Data Availability

The data used to support the findings of this study are available from the corresponding author upon request.
